# Evaluation of fit and fracture strength of three-dimensional provisional crowns printed with different orientations and different layer thicknesses: an in-vitro study

**DOI:** 10.1186/s12903-025-07330-2

**Published:** 2025-12-10

**Authors:** Gehad Mohamed Ali Ibrahim, Tarek Abdelhamid Abdelhamid, Mosaad Ali Elgabrouny, Amr Abdelaziz Shebl Kassem

**Affiliations:** 1 Assistant lecturer in Fixed Prosthodontics Department, Suez Canal University, Ismailia, Egypt; 2 Associate Professor of Fixed Prosthodontics Department, Suez Canal University, Ismailia, Egypt; 3 Professor of Fixed Prosthodontics Department, Suez Canal University, Ismailia, Egypt; 4 Associate Professor & Chairman of Fixed Prosthodontics Department, Faculty of Dentistry, Suez Canal University, Ismailia, Egypt

**Keywords:** 3D printing, Provisional crowns, Printing orientation, Layer thicknesses, Stereomicroscope, Absolute marginal discrepancy (AMD), Fracture resistance

## Abstract

**Aim:**

The effect of 3D printing parameters on the marginal fit and fracture resistance of 3D printed provisional crowns is not clear. The objective of this research was to use different printing orientations and layer thicknesses to build 3D printed provisional crowns and to evaluate the marginal fit and fracture resistance of it.

**Materials and methods:**

A maxillary first premolar resin tooth was reduced for all-ceramic tooth preparation then was scanned using intraoral scanner. 60 dies were printed using Digital Light Processing (DLP) 3D printer. One of the printed dies was scanned. The design of the crown was made then the design was exported to 3D printing software. 60 provisional resin crowns were printed using DLP 3D printer. 36 Provisional crowns were divided into 3 groups(*n* = 12) according to the printing orientation used: group A (0°), group B (90°) and group C (30°)0.24 Provisional crowns were divided into 2 groups (*n* = 12) according to the layer thickness used: group D (50 μm) and group E (100 μm). Absolute marginal discrepancies (AMD) were measured using stereomicroscope. Fracture resistance was measured using a universal testing machine. Data analysis was performed using one way-ANOVA, independent T- test and paired t-test at 0.05 using IBM SPSS version 26.0.

**Results:**

One-way ANOVA revealed a significant effect of printing orientation and layer thickness on the marginal fit and fracture strength of 3D printed crowns (p < 0.001). One-way ANOVA showed that group C (60.76 [± 6.96]μm) showed significantly lower mean [± SD] absolute marginal discrepancy (AMD) in µm than group A (72.26 [± 6.07] μm) and there was no statistically significant difference between group B and group C. One-way ANOVA revealed that there was a statistically significant difference in the mean value of fracture resistance between group B and each of two other groups: group A and group C. The highest mean value [± SD] was reported with the samples of group A (977.97 [±10.69]), while the lowest mean value was reported with the samples of group B (785.10 [± 20.43]) (p < 0.001).

**Conclusion:**

Printing orientation and layer thickness influenced marginal fit and fracture resistance of 3D printed provisional crowns.

## Introduction

Temporary restorations play an essential role in the field of fixed prosthodontics rehabilitation, especially when extensive treatment is required before the final prosthetics can be completed [1].

Provisional restoration may be defined as a fixed or removable dental or maxillofacial prosthesis intended to restore function and aesthetics for a brief amount of time before being replaced by a final prosthesis. The provisional restorations are essential to protect the prepared teeth from thermal, chemical and physical irritations and restore esthetics, phonetics and chewing function in addition to fixing the teeth position until the final restorations are cemented [2].

The materials used for the temporary crown or bridge must satisfy certain requirements related to their mechanical, biological, and aesthetic qualities. It should provide protection to pulp from thermal and chemical irritation after tooth preparation. It should provide function, esthetic and preserve the health and the shape of gingiva, so, it should have a highly polished surface to facilitate plaque removal and maintain a healthy periodontium. It should withstand force of mastication during the period of its use [3].

One of the most ideal requirements of a proper provisional restoration is the marginal seal. The margin of the restoration should provide good protection for the prepared tooth in addition to its gingival tissues which is necessary for further cementation. Marginal discrepancies may lead to microleakage which is responsible for post operative sensitivity and recurrent caries [1].

The provisional restorations can be classified according to the method of fabrication into direct(chairside), indirect (in the laboratory) and indirect- direct. Also, they can be classified according to the duration of its use into short term restoration (which can be used for several days to week), medium term restoration (which can be used for several weeks) and long-term restoration (which can be used for several months) [4].

Indirect fabrication methods of the provisional restorations include subtractive manufacturing and additive manufacturing. Subtractive manufacturing using CAD-CAM technology fabricates the provisional and the final fixed dental prostheses based on the same data set [5]. But most of the material is wasted when the provisional restoration is milled [6].

Additive manufacturing using 3D printing technology had rapidly developed in recent years, because of its reliability and accuracy, making it highly attractive to the medical field like dentistry, medicine, engineered tissue models and orthopedics [7, 8].

Different additive manufacturing techniques are available as Powder Bed Fusion, light curing, Binder Jetting, and Fused Deposition Modeling. These techniques mainly differ in the material used and the way the layers are built to produce the 3D object [9].

Light curing technology is a broad term used to describe a specific type of 3D printing methodology that involves the use of photosensitive resin materials that undergo curing and shaping when exposed to light irradiation [10]. This technology involved three main methods: Stereolithography, digital light processing, and photo jet. Stereolithography is a widely used method of rapid prototyping in which a laser cures layers of light-sensitive polymer in a liquid polymer tray [11]. Stereolithography is favored for its ability to produce highly accurate prototypes with complex shapes, strong mechanical properties, and a smooth surface finish. However, there are some drawbacks including: the high cost of equipment, the need for post-curing, time consuming and the limitation to using only polymers [12, 13].

Digital Light Processing (DLP) involves exposing a tray of liquid resin to intense light from a projector, selectively solidifying the resin onto a building platform in a sequential, layer-by-layer manner. DLP technology enables the simultaneous projection of an entire layer of a 3D model, curing all points simultaneously [14]. DLP printers are known for their efficiency and speed compared to Stereolithography (SLA) printers due to their use of DMDs (a digital micro-mirror display). Unlike SLA printers that utilize guided lasers, DLP printers can solidify all points in a layer simultaneously, resulting in improved overall efficiency [15].

The advantage of DLP over SLA is fast the fabrication of the printed layers through printing and curing a single layer across the total build plate in few seconds, thus decreasing the cost of production [16–18].

Commonly, the printing process is an automated process in which some parameters are pre-set like energy distribution, cure depth, printing velocity and cured line width. But some preprocessing parameters can be adjusted like layer thickness, build angle and object positioning on the build platform [19].

The mechanical properties of 3D printed restoration are not only influenced by the material but also by the manufacturing process. It may be influenced by the printing orientation which may lead to different mechanical behavior of the fabricated product depending on the build angle [20].

According to what was mentioned above it was found interesting to assess the effect of changing printing angle and layer thickness on marginal fit and fracture strength of 3D printed provisional restoration using DLP technology. The null hypothesis was: There was no significant effect of changing printing orientation and layer thickness on the marginal fit and the fracture strength of provisional crowns printed with DLP 3D printer.

## Materials and methods

### Study design

This study was carried out after approval of Research Ethics Committee (REC), Faculty of Dentistry, Suez Canal University (approval number 577/2022).

### Sample size calculation

One-way analysis of variance was proposed (ANOVA). The sample size was calculated according to G*Power software version 3.1.9.2. Accordingly, a total sample size of 60 were found to be sufficient to detect the effect size of 0.85, a power (1-β) of 75% and at a significant level of 5% (*p* < 0.05), with 12 samples for each group. The sample size in this study was in agreement with Faul et al. 2007 [21].

### Samples Preparation and grouping

#### Printing the dies

A maxillary first premolar resin tooth (Nissin Dental, Kyoto, Japan) was reduced by 2 mm preparation on the occlusal surface and a total of 6° convergence angle with a 1 mm circumferential deep chamfer finish line. Then, it was scanned using intraoral scanner (Omnicam, Dentsply Sirona, United States). The 3D virtual model of the scanned tooth was sent in STL format (Standard Tesselation Language) to Accuware 3D printing software program of DLP 3D printer (AccuFab-D1s, Shinning 3D, China) which has an XY resolution accuracy of 50 μm and a layer thickness capability ranging from 25 to 100 μm, as mentioned by the manufacturer.

60 resin dies were printed using DLP resin (NextDent Model, Vertex-Dental, Netherlands). The 3D printing parameters were determined using 3D printing software as follows: layer thickness 50 μm and horizontal printing orientation (0°). Then, cleaned using an ultrasonic activated bath of 90% isopropyl alcohol for 5 min. Printed dies were post cured in the post curing unit (Fab Cure. Shinning 3D, China) with wavelengths 385 to 405 nm for about 20 min according to the manufacturer’s instructions.

#### Printing the crowns

For samples of this study to be identical, one of the printed dies was scanned using the intraoral Omnicam scanner. Then, the 3D virtual model of the scanned die was sent in STL format to the CEREC InLab 16 software for crown designing.

The cement space was set to 60 μm. The design was exported as a STL file to 3D printing software program (Accuware, Shinning 3D, China).

A total of 60 provisional crowns were printed using DLP resin (NextDent temp C&B, Vertex-Dental, Netherlands). The 3D printing parameters including (printing orientation, layer thickness) were established in the software according to each printed group (*n* = 12).

**Fig. 1:**
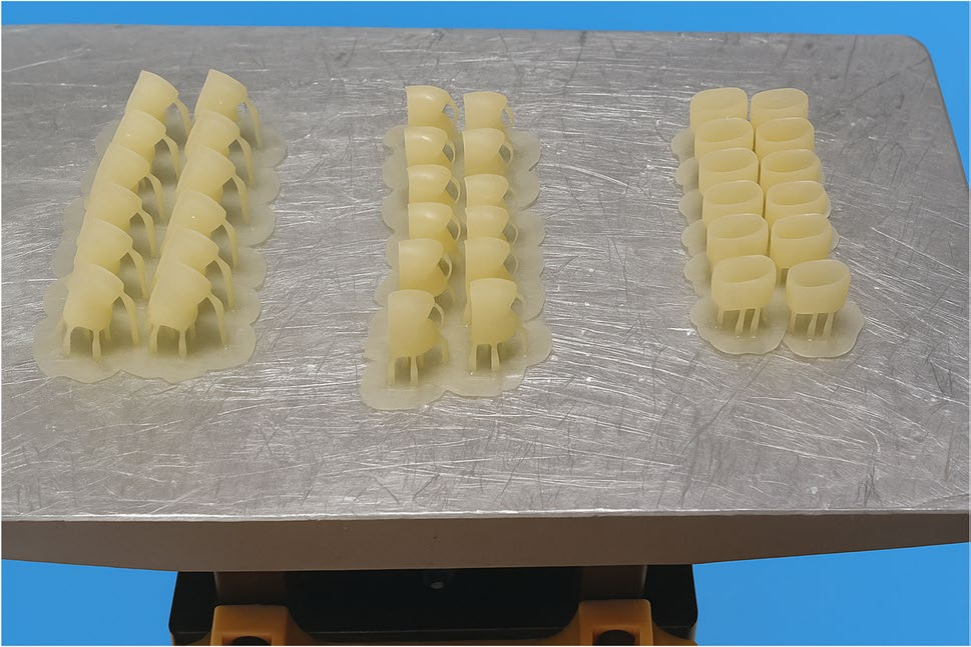
3D printed crowns with different printing orientations on the building platform (Groups A, B and C)

**Fig. 2:**
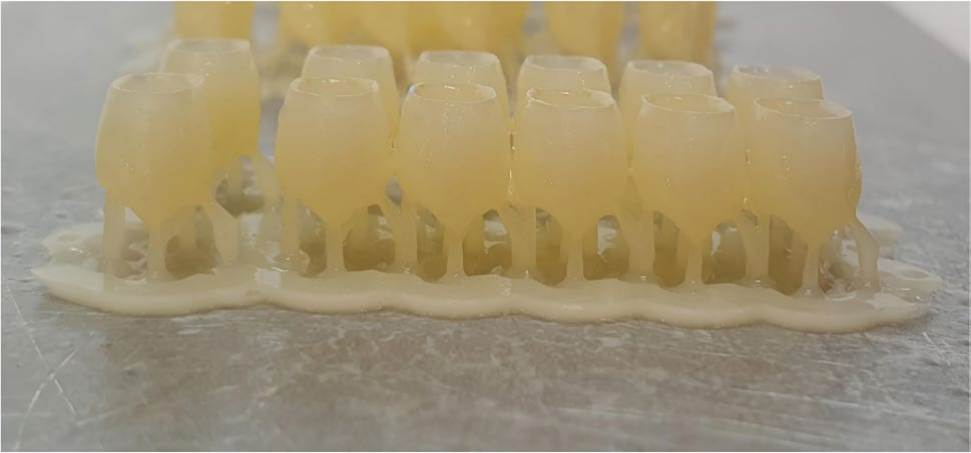
Crowns printed with horizontal orientation (Group A, 0° (occlusal surface facing platform)

**Fig. 3:**
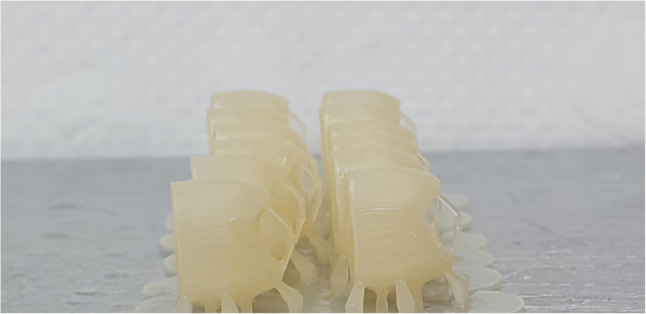
Crowns printed with vertical orientation (Group B ,90°) (buccal surface facing platform with 90°)

**Fig. 4:**
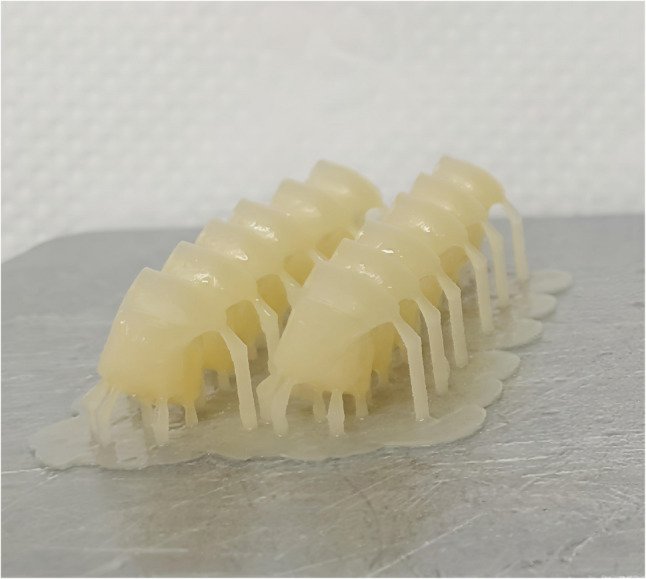
Crowns printed with diagonal orientation (Group C, 30° (buccal surface facing Platform with 30°)

For groups A, B, C the provisional crowns were printed with the same layer thickness 50 μm and the different printing orientations as follow Figure 1: group A (*n* = 12) 0° (horizontal orientation, occlusal surface facing the building platform) Figure 2, group B(*n* = 12) 90° (vertical orientation, buccal surface facing the building platform and making angle 90°with it) Figure 3 and group C 30° (diagonal orientation, buccal surface facing the building platform and making angle 30°with it) Figure 4.

Crowns were later evaluated for absolute marginal discrepancy (AMD) and fracture resistance on their corresponding dies.Group C showed the most favorable results in the performed tests. Therefore, diagonal printing orientation (30°) was used as the printing orientation for the remaining groups.

For groups D, E the provisional crowns were printed with the same printing orientation 30° and the different layer thicknesses: (group D(*n* = 12) 50 μm, group E(*n* = 12) 100 μm)

**Fig. 5:**
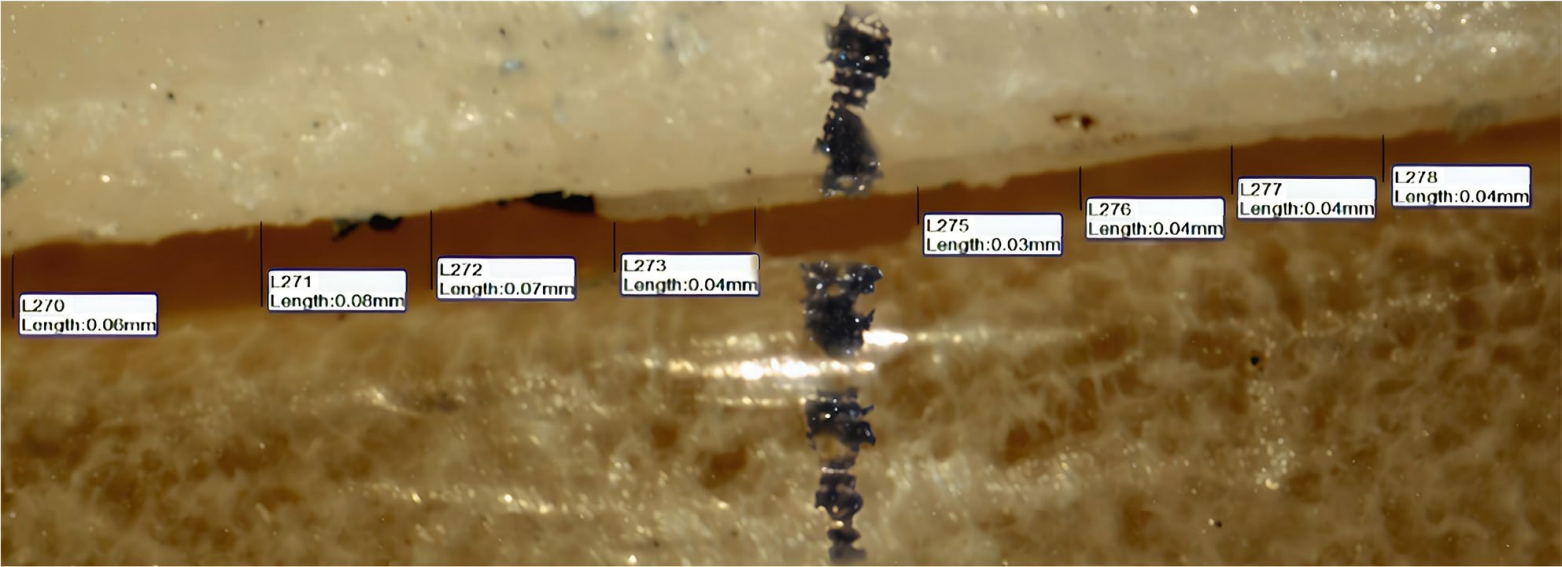
The absolute marginal discrepancies (AMD)of group A (horizontal printing orientation 0°) sample under a stereomicroscope on buccal surface

**Fig. 6:**
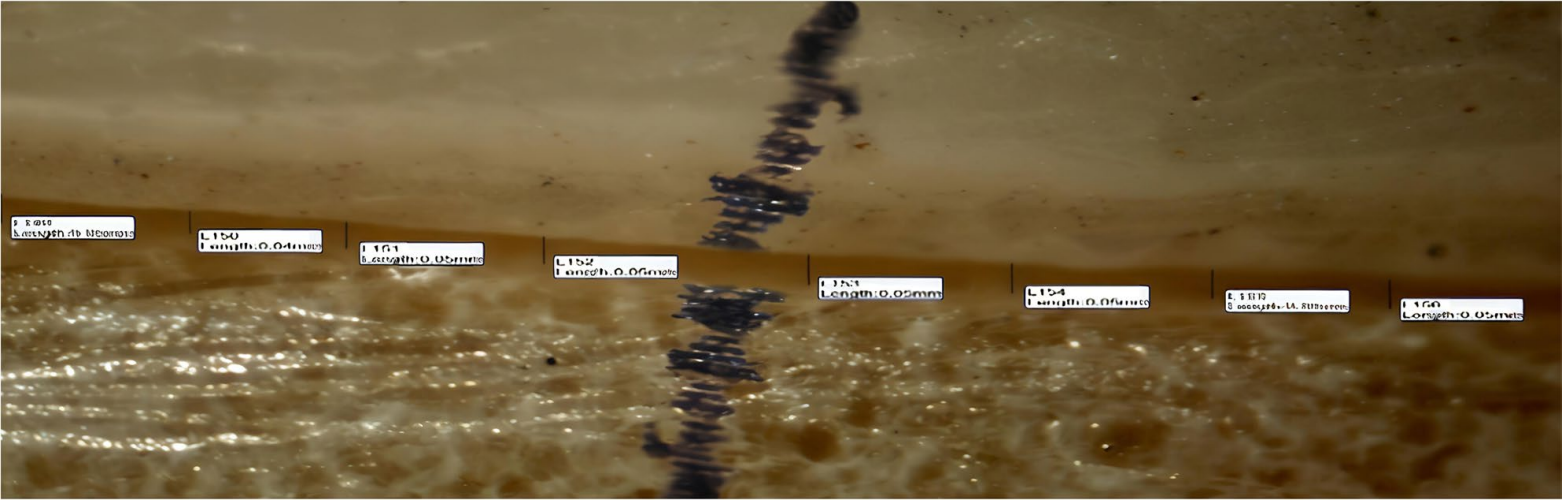
The absolute marginal discrepancies (AMD) of group B (vertical printing orientation 90°) sample under a stereomicroscope on buccal surface

**Fig. 7:**
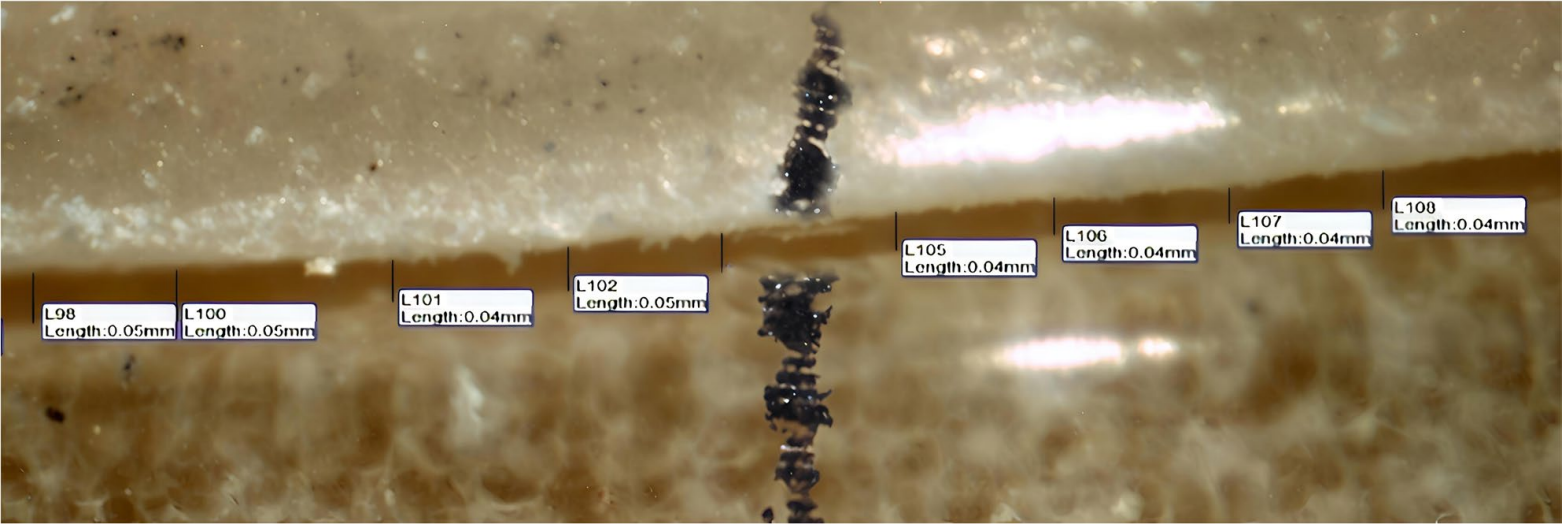
The absolute marginal discrepancies (AMD)of group C (diagonal printing orientation 30 °) sample under a stereomicroscope on buccal surface

Absolute marginal discrepancy (AMD was assessed by measuring the vertical distance between two specific points, one located on the crown margin and the other on the die finish line parallel to the tooth axis. Each surface of the crown (buccal, lingual, mesial, and distal) was divided into two equal halves(midbuccal, midlingual, midmesial, middistal), and four measurement points were taken from each half, resulting in eight points per surface as shown in Figures 5, 6 and 7 and a total of 32 readings per crown. This subdivision was applied to try to obtain standardization and even distribution of measurement sites across all crown surfaces, minimizing variations and enhancing measurement reliability. The mean value of the 32 readings was recorded as the final absolute marginal discrepancy (AMD) for each specimen.

A snapshot for the absolute marginal discrepancy (AMD) was taken with a high-resolution digital camera mounted on the Olympus stereomicroscope (Olympus, Japan). A software module (IS Capture) was used to analyze the images. The measurements were taken at a magnification of 35 X. All the imaging was taken. The measurements in the four surfaces together (buccal, mesial, lingual, and distal) were measured and taken as the final mean for each sample.

The printed crowns were cemented onto the printed dies with temporary non eugenol cement (Charm Temp NE, Dentkist, South, Korea). To ensure that the cement thickness had no effect on the results, a special cementing device with a defined pressure was used which allowed static placement of 3Kg load on the occlusal surface of the crowns during the cementation procedures.

Fracture resistance was measured for crowns of each group were individually mounted on a computer controlled universal testing machine (Tyra, United States). The sample was fixed on the machine using a special holding device through its screws. This device was attached to the lower fixed compartment of the universal testing machine. Fracture test was done by compressive mode of a load applied occlusally using a steel indentor with 5.6 mm diameter spherical tip at a crosshead speed of 1 mm/min with a tin foil sheet 0.5 mm thickness in between to achieve homogenous stress distribution and to avoid contact damage of the steel indenter.

### Statistical analysis

All data were collected, calculated, tabulated, and statistically analyzed using Shapiro-Wilk and analyzed using One-way ANOVAs, Bonferroni’s post hoc test Dependent (paired) and Independent T- test. All Statistical analysis was performed using the computer program SPSS software for windows version 26.0 (Statistical Package for Social Science, Armonk, NY: IBM Corp) at a significance level of *p* ≤ 0.05.

## Result

The mean ± standard deviation (SD) for groups (A, B and C) in µm are listed in Table 1. One way ANOVA revealed a significant effect of printing orientation on the absolute marginal discrepancy (AMD) of 3D printed crowns (*p* < 0.001). Regarding printing orientation, one-way ANOVA showed a statistically significant difference between group A and group C. Group A showed the highest mean value of the absolute marginal discrepancy (AMD) (78.78 [± 4.05] µm). Group C showed the lowest mean value of AMD (70.33 [± 3.47] µm) and there was no statistically significant difference with group B (72.92 [± 5.90] µm) (*p* < 0.001).

**Table 1:** Mean and standard deviation (SD) values of marginal gap for groups A, B,C

Groups	Mean	SDP value
Group A	78.78^a^	4.05 < 0.001**
Group B	72.92^b^	5.90
Group C	70.33^b^	3.47

Mean and standard deviation (SD) values of AMD recorded in µm after cementation for the three groups (A, B, C) printed with different printing orientations

** and different letters (a, b) mean significant difference using one way ANOVA test at *p* < 0,0.05

The mean ± standard deviation (SD) fracture resistance values for groups A, B and C were determined after cyclic loading and are presented in Table 2. Group A showed the highest statistically significant fracture resistance mean value (977.97[± 10.69]N), followed by group C (967.14[± 15.03]N), while the lowest statistically significant fracture resistance mean value was recorded for the group B (785.10 [± 20.4]N) (*p* < 0.001) and there was no statistically significant difference between group A and group C.

**Table 2:** Mean and standard deviation (SD) values of fracture resistance for groups A, B, C

Groups	Mean	SD	*P* value	
Group A	977.97^a^	10.69		<0.001**
Group B	785.10^b^	20.4		
Group C	967.14^a^	15.03		

Mean and standard deviation (SD) values of the fracture resistance recorded in newton for the three groups (A, B,C) printed with different printing orientations.

** and different letters(a, b) mean significant difference using one way ANOVA test at *P* < 0,0.05

The mean ± standard deviation (SD) for groups (D and E) in µm are listed in Table 3. The independent T test revealed a significant effect of layer thickness on the absolute marginal discrepancy (AMD) of 3D printed crowns (*p* < 0.05). Regarding layer thickness, independent T test showed a statistically significant difference between group D and group E. Group E showed the highest mean value of absolute marginal discrepancy (AMD) ([82.72 ± 2.96] µm). Group D showed the lowest mean value of the absolute marginal discrepancy (AMD) (70.33 [± 3.47] µm) (*p* < 0.05).

**Table 3:** Mean and standard deviation (SD) values of absolute marginal discrepancy (AMD) for the groups D, E

Groups	Mean	SD	*P* value
Group D	70.33	3.47	<0.001**
Group E	82.72	2.96	

Mean and standard deviation (SD) values of the absolute marginal discrepancy (AMD) recorded in µm after cementation for the two groups (D, E) printed with different layer thicknesses: group D (50 μm) and group E (100 μm)

** means significant difference using independent T test at *p* < 0.05

The mean ± standard deviation (SD) fracture resistance values for groups D and E were determined after cyclic loading and are presented in Table 4. Independent T test at *p* < 0.05 showed that there was no statistically significant difference between group D (956.97{±15.35} N) and group E (967.50{±14.49}N).

**Table 4:** Mean and standard deviation (SD) values of the fracture resistance for group D, E

Groups	Mean	SD	*P* value
Group D	956.97	15.35	>0.05
Group E	967.50	14.49	

Mean and standard deviation (SD) values of fracture resistance recorded in newton for the two groups printed with different layer thicknesses group D (50 μm) and group E (100 μm). *p* > 0.05 means no significant difference using independent T test at *p* < 0.05

## Discussion

This study was performed to evaluate the effect of 3D printing orientations and layer thicknesses of 3D printed provisional crowns on marginal fit and fracture resistance on the upper 1 st premolar. Depending on the result, the marginal fit is affected by different printing orientations and different layer thicknesses. Fracture resistance was affected by changing printing orientation. The null hypothesis was rejected depending on the finding of the comparative of marginal fit and fracture resistance for the different printing orientations and layer thicknesses.

All dies were printed and post cured using the same 3D printer, building platform, resin type, printing parameters (50 μm layer thickness and horizontal orientation) and printing at the same time. Any possible dimensional variation caused by printing or post curing process would have affected all samples equally. Therefore, the dies can be considered identical in terms of fabrication parameters.

Based on earlier research, standardization of the cement space of the crowns was achieved by selecting 60 μm cement space on software [22].The design was exported as a STL file to Accuware 3D printing software program to ensure that 60 crowns were printed with the same design changing in 3D printing parameter according to each printed group. The fabrication and post-curing processes were made by a single trained practitioner to eliminate human variations.

The printed crowns were cleaned using an ultrasonic activated bath of 90% isopropyl alcohol for 5 min to remove the uncured resin [23].Then, the crowns were placed in post curing unit to cross link unreacted monomers which were presented due to the oxygen in the air diffusing into the outer layers of resin while printing, preventing the printed object from completely curing. So, post curing was an essential step to complete the polymerization process of the printed crowns, thus improving their mechanical properties[7, 24]. For measurement of the absolute marginal discrepancy (AMD) of the crowns, Olympus stereomicroscope (Olympus, Japan) was used due to its accuracy and non-invasiveness [22].A software module (IS Capture, Radical, India) was used to analyze the images.

To standardize the production of provisional crowns, all crowns were printed using the identical STL file and layer thickness of 50 μm as mentioned by the manufacturer, with modification of printing orientation according to each group.

The number and distribution of the measurement points of absolute marginal discrepancy (AMD) (32 readings for each crown) used in the present study were consistent with those reported by the previous studies Groten et al. (2000)[25] and Ozkurt-Kayahan et al. (2024) [26].

Group C printed with 30° orientation showed the lowest absolute marginal discrepancy (AMD). This might be attributed to the fact that with this printing orientation, the buccal surface of the crown was positioned at an angle of 30° facing the printing platform. Polymerization shrinkage was directed towards the buccal surface, thereby avoiding margin distortion. Also, this might be attributed to the fact that polymerization shrinkage occurred towards areas with higher resin content. Areas connected to supporting structures might show large amount of polymerization shrinkage as they contain high amount of resin volumes compared with other areas during printing [24]. As long as the amount of resin increased, the polymerization shrinkage also increased as mentioned byPrasad K et al. [3]. On the other hand, when the printing orientation was horizontal (0°) in group A, the 3D printed samples exhibited the highest average values of the absolute marginal discrepancy (AMD). This might be attributed to the fact that with this orientation, the occlusal surface of the crowns faced the 3D printer platform, where it was connected to the supporting structure with high resin volume in that area. This might lead to polymerization shrinkage directed towards this surface, resulting in larger absolute marginal discrepancy (AMD). This came in agreement with a similar study by G S Park et al. [27].

Osman et al. [28] investigated the dimensional accuracy of the provisional crowns in 9 different angles using a DLP 3D printer. The result showed that 135° offered the highest dimensional accuracy and the most favorable deviation pattern followed by 150° which is considered corresponding to 30° in the present study. The build angle 270° which corresponds to build angle 90° showed no significant difference with 150°.

Ryu et al. [29] investigated the marginal and internal fit of 3D printed provisional crowns using 6 building directions. They reported that building angle 150° which corresponds to 30° build angle in the current study had the best fit.

Also, Park et al. [27] evaluated the effect of printing parameters on the fit of implant-supported 3D printing resin prosthetics. The dental model was replicated and produced for three-unit prostheses with two implants. The result showed that there was a significant difference in the marginal and internal fit with different building angles and layer thicknesses and concluded that optimal build angle was 45° and 60°and the optimal layer thickness was 50 μm. The findings of this study were particularly inconsistent with the present study which mainly related to the effect of building orientation. This might be attributed to the variation in resin used and the discrepancy between the restoration categories, including FPDs and individual crowns supported by teeth or implants.

Yang et al. (2022) [24] evaluated the effects of build orientations and layer thickness on the marginal fit and absolute marginal discrepancy of 3D-printed three-unit fixed partial dentures (FPDs). Their findings reported that variations in printing thickness did not significantly influence the marginal fit of the restorations. The findings of this study are not in agreement with the results of the present study. This discrepancy may be attributed to several factors, including differences in the type of material used, differences in the measurement methodology, experimental conditions and the differences in the types of restorations (FPDs versus single crowns on implants or teeth abutments).

Revilla-León et al. (2025) [30] reported that 0° printing orientation showed the highest trueness and precision and 90° printing orientation showed the lowest trueness and precision which was not agreement with the present study. This might be attributed to variation in 3D printing technology (using SLA) and different types of resin.

The results of fracture resistance test in the present study were significantly influenced by both printing orientation and loading procedure applied. Group (A) showed the highest mean value of fracture resistance. This might be attributed to the alignment of the layers during printing in relation to the applied force. In the horizontal printing orientation (0°), the provisional crowns were printed with the occlusal surface facing the building platform. So, the force applied in a vertical direction on the center of occlusal surface of the crown was perpendicular to the crown layers resulting in high fracture resistance values [23].While in group B, the lowest mean value of fracture resistance was observed. The provisional crowns were printed with the buccal surface facing the building platform at a 90° angle, causing the applied forces to be parallel to the interfaces between the layers leading to stress concentration and separation. This could result in low fracture resistance values [31]. Furthermore, group C showed no statistically significant difference in the mean value of fracture resistance with group A. The provisional crowns were printed with the buccal surface facing the building platform at a 30° angle. The applied force was directed oblique on the layers that might lead to analysis of this force into destructive and non-destructive force. The effect of destructive force would be decreased, which is parallel to the layers as reported byDiken Turksayar et al. [32]. The result of the present study was in agreement with Alharbi et al., Diken Turksayar et al. and Nold et al. [31, 32, 23].

As, group C showed the most favorable results in comparison to groups A and B in the tests performed. Therefore, the diagonal printing orientation (30°) was used as the printing orientation for printing of the remaining groups (D, E). To standardize the printing process for crowns of groups (D, E), all crowns were printed using the same STL file and were oriented at a set angle of 30° that was selected based on the achieved outcome. The only variation between groups was in the layer thickness, which was adjusted according to each specific group. Group D showed the lowest mean value of absolute marginal discrepancy (AMD). This could be explained by the fact that the most difficult areas to reproduce in fixed dental prothesis are the thin areas such as complex geometrical margins. Therefore, a small printing layer thickness can help to ensure that all the fine details are accurately reproduced, especially in margins [33]. Another cause might be related to the principles of DLP technology as light-cured resin is applied layer by layer. However, in cases where non-linear borders such as the provisional crowns in the present study are present in the printed design, these layers are not directly aligned on the z-axis or xy-plane but in the form of discrete points. The thickness of each layer directly impacted the number of discrete points on the edge of the printed object. A thinner layer (50 μm layer thickness) might create more discrete points, resulting in a smoother and more detailed surface, ultimately improving the accuracy of the printed object [34]. While with group E, 100 μm layer thickness might be too thick to accurately reproduce thin areas like margins. As, thicker layers of material might lead to loss of fine details in the final output [33]. Also, thick layer thickness produces fewer discrete points with greater distances between them, leading to a noticeable stair-stepping effect [34]. The result of the current study came in agreement with Çakmak et al. (2022) [33], Hasanzade et al. (2023) [35], Sabbah et al. (2021) [36], Yang et al. (2022) [24] and Zhang et al. (2019) [34].

There was no statistically significant difference in the mean value of fracture resistance between group D and group E. If the layer thickness increased, the number of layers decreased. Decreasing number of layers might lead to decrease cumulative error that might lead to improved mechanical properties as reported by Hasanzade et al. (2023)[35]. That is why 100 μm layer thickness showed the high mechanical properties.

Aljehani et al. (2024) [37] evaluated the effect of three different printing orientations (0°,45°,90°) on the fracture resistance and marginal quality of 3D-printed anatomic provisional crowns. They reported that the printing orientation of 0° showed the best marginal quality, while printing at 90° showed the highest fracture resistance. The result of this study was not in agreement with the results of the present study. These variations might be attributed to the different materials used and the different conditions.

Özden et al. (2025) [38] reported no significant effect of different printing orientations (0°,90°) on flexure strength of 3D-printed materials, while aging significantly reduces flexure strength, particularly after simulating three years of intraoral use. 3D-printed resins reach stability more quickly under aging conditions compared to micro hybrid composites, while milled resins continue to exhibit superior mechanical properties. That long-term mechanical stability is more substantially compromised by hydrolytic degradation from thermal aging than by the initial build parameters of print orientation. While the present study found that different printing orientations showed significant effect on fracture strength. This discrepancy in results might be attributed to the differences in the resin composition used in the studies and the difference in post-processing protocols (e.g., washing, curing parameters), which significantly affect the degree of conversion and the final material properties.

The clinical relevance of the obtained results should be interpreted in the context of previously reported acceptable thresholds for provisional restorations. According to the literature, Yao et al. (2014)[39], Ryu et al. (2020)[29], and Beuer et al. (2009)[40] reported the range of marginal gap in provisional crowns 150–280, 58–113, and 100–150 μm, respectively. Marginal gaps below 120 μm are considered clinically acceptable for provisional crowns. In the present study, the mean (AMD) values ranged between 65 and 78 μm, which are well within this clinically acceptable range. Similarly, fracture resistance values exceeding 300–500 N are sufficient to withstand normal masticatory forces in provisional restorations. The recorded values in this study ranged between 700 and 900 N, exhibiting proper mechanical properties under clinical conditions. These findings came in agreement with(Diken Turksayar et al. (2022) [32] confirm the potential clinical applicability of the tested printing parameters for provisional restorations.

### Limitations

One of methodological limitations in the present study was using Omnicam, intraoral scanner (Dentsply Sirona, USA). As it was less advanced than newer scanner technologies and might have influenced the accuracy of digital impressions. Therefore, it is recommended that future research employ more advanced intraoral scanner technologies to enhance accuracy and reliability of digital measurements. Another methodological limitation of this study was the use of only DLP printing technology. Future research should consider other techniques, such as SLA which might show different results.

## Conclusion

Within the limitations of this in-vitro study, it can be concluded that changing printing orientation and layer thickness significantly affect the marginal fit and fracture resistance of 3D-printed provisional crowns. The diagonal printing orientation (30°) and the layer thickness (50 μm) showed the most favorable results. The obtained absolute marginal discrepancy (AMD) and fracture resistance values were within clinically acceptable limits, suggesting that these parameters can provide reliable and durable provisional restorations. Further in-vivo studies are recommended to validate these findings under clinical conditions. Future studies are recommended to evaluate different 3D printing technologies, orientations, and resin materials to better understand their effects on the accuracy and strength of printed provisional restorations.

## Data Availability

The data sets generated and analyzed during this investigation are not publicly available due to (ownership of data); however, they are available from the corresponding author upon reasonable request.
